# Advanced Differentiated Thyroid Cancer: A Complex Condition Needing a Tailored Approach

**DOI:** 10.3389/fonc.2022.954759

**Published:** 2022-07-07

**Authors:** Antonio Mario Bulfamante, Eleonora Lori, Maria Irene Bellini, Elisa Bolis, Paolo Lozza, Luca Castellani, Alberto Maria Saibene, Carlotta Pipolo, Emanuela Fuccillo, Cecilia Rosso, Giovanni Felisati, Loredana De Pasquale

**Affiliations:** ^1^ Otolaryngology Unit, ASST Santi Paolo e Carlo, Department of Health Sciences, University of Milan, Milan, Italy; ^2^ Department of Surgical Sciences, “Sapienza” University of Rome, Rome, Italy; ^3^ University of Milan, Milan, Italy; ^4^ Thyroid and Parathyroid Surgery Service-Otolaryngology Unit, ASST Santi Paolo e Carlo, Department of Health Sciences, University of Milan, Milan, Italy

**Keywords:** thyroid, thyroid cancer, papillary carcinoma, follicular carcinoma, differentiated carcinoma, thyroidectomy, lymph node dissection

## Abstract

Differentiated thyroid cancers (DTCs) are slow-growing malignant tumours, including papillary and follicular carcinomas. Overall, prognosis is good, although it tends to worsen when local invasion occurs with bulky cervical nodes, or in the case of distant metastases. Surgery represents the main treatment for DTCs. However, radical excision is challenging and significant morbidity and functional loss can follow the treatment of the more advanced forms. Literature on advanced thyroid tumours, both differentiated and undifferentiated, does not provide clear and specific guidelines. This emerges the need for a tailored and multidisciplinary approach. In the present study, we report our single-centre experience of 111 advanced (local, regional, and distant) DTCs, investigating the rate of radical excision, peri-procedural and post-procedural complications, quality of life, persistence, recurrence rates, and survival rates. Results are critically appraised and compared to the existing published evidence review.

## Introduction

Thyroid cancer accounts for less than 1% of all malignancies, but for about 5% of all thyroid nodules (1). Among thyroid cancers, differentiated thyroid carcinoma (DTC) is the most common and includes papillary thyroid cancer (PTC), follicular thyroid cancer (FTC), and Hürthle cell thyroid carcinoma (HTC) (2). DTC has an annual incidence of 1/10,000 with a female-to-male ratio of 3:1 (3, 4). DTC incidence is increasing, most probably due to the continuous improvement offered by early diagnostic tools (4–6). DTCs have a favourable prognosis, with a 5-year survival rate of approximately 93% and 88% in women, and men, respectively (4), although unfortunately, 5% of DTCs are fatal (3). Surgery represents the initial treatment of choice (7, 8), followed in most cases by radioactive iodine (RAI) I^131^ to eradicate microscopical disease or distant metastases. In addition, multikinase inhibitors (MKIs), including sorafenib and lenvatinib, are rising as alternative medical treatments in the most advanced forms, especially in RAI-refractory differentiated thyroid cancer (RR-DTC). Advanced disease accounts for 13%–15% of DTCs and is characterized by a worse prognosis and more challenging management (5). Advanced DTC (ADTC) is defined by the existence of one or more of the following conditions: local invasion, bulky cervical nodes, and/or distant metastases. A recent review by Russel et al. describes all the issues associated with advanced thyroid cancer, from the lack of adequate guidelines and the need for a multidisciplinary and tailored approach. This is necessary to overcome the challenges of the distinct DTC subtypes, such as those related to surgical radicality, meaning not only total thyroidectomy and lymphadenectomy, but even tracheal ring resection, partial or total laryngectomy, or thoracic access. Based on the aforementioned study (4), the aim of the present manuscript is to critically review our single-centre experience of advanced thyroid cancer (local, regional, and distant), but focusing specifically on ADTC, thus not dealing with medullary, anaplastic carcinoma, or other undifferentiated cancers, because of their worst prognosis and the limited therapeutic options and surgical strategies currently available. Herein, we provided our DTC 30-year experience on therapeutic approach, peri-procedural and postsurgical management, quality of life disease recurrence, and survival rates.

## Materials and Methods

A retrospective analysis of medical and electronic charts of a single-centre series comprised 656 thyroid tumours, 627 of which are DTCs (333 PTC, 92 FTC, 202 papillary microcarcinoma), 21 medullary thyroid carcinomas, 1 squamous cell carcinoma, and 7 anaplastic tumours. An exception from Institutional Review Board evaluation was granted due to the study’s retrospective nature. Our experience herein reported involved 111 consecutive patients diagnosed with ADTC who underwent a surgical assessment between May 1992 and September 2021 at the Outpatient Endocrine Surgery Service, Thyroid and Parathyroid Surgery Unit, in San Paolo Hospital, Milan, Italy. Before surgery, our protocol included blood tests, with particular attention to the TSH, calcium, PTH, 25 – OH vitamin D, and calcitonin blood levels. Neck ultrasound was always the first imaging examination, to evaluate for thyroid nodules and cervical lymph nodes. All patients with ultrasonographical suspicion of nodules and/or lymph node involvement underwent ultrasound-guided fine-needle aspiration (FNA) to confirm DTC cytologically. In case of suspicion of infiltration of surrounding tissues, further targeted radiological examinations, such as neck and chest CT, were performed.

**Figure 1:**
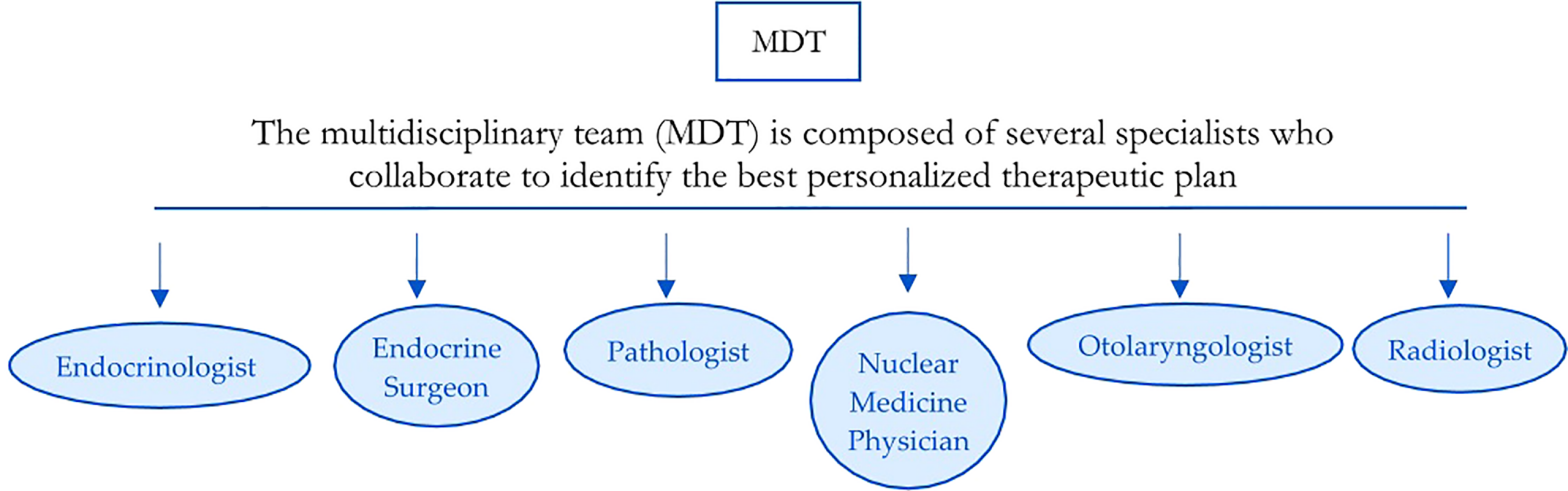
Multidisciplinary team.

All patients underwent a preoperative evaluation with video-laryngoscopy by an expert otolaryngologist. All ADTC cases were discussed pre- and postoperatively by a multidisciplinary team (MDT) consisting of an endocrinologist, an endocrine surgeon, an otolaryngologist, a pathologist, a radiologist, and a nuclear medicine physician (**Figure 1**).

Risk factors in the patient’s medical history, such as family and personal history of thyroid disease, cancer, goitre endemics, and radiation exposures, were also analysed. Symptom onset was identified when the presence of local complaints such as dysphagia, dysphonia, dyspnoea, sensation of cervical encumbrance and cervical dysmorphism, lymphadenopathies, or distant metastases appeared. Patients were divided into the following classes according to the surgery performed: total thyroidectomy with adjacent structure resection and centra cervical lymphadenectomy (TT + CCL), total thyroidectomy and central and unilateral cervical lymphadenectomy (TT + CUCL), ;total thyroidectomy and central and bilateral cervical lymphadenectomy (TT + CBCL), unilateral cervical lymphadenectomy (UCL), and bilateral cervical lymphadenectomy (BCL) after previously performed thyroidectomy. “Cervical lymphadenectomy” was intended as modified radical neck dissection, including II, III, IV, and V levels. Only five patients, underwent additional resection of distant metastases. We systematically started the use of intraoperative inferior laryngeal nerve neuromonitoring in 2018. We also analysed the rate of complications and divided them into transient hypocalcaemia (TH), persistent hypocalcaemia (PH), recurrent laryngeal nerve transient unilateral palsy (RLNTUP), recurrent laryngeal nerve transient bilateral palsy (RLNTBP), recurrent laryngeal nerve persistent unilateral palsy (RLNPUP), recurrent laryngeal nerve persistent bilateral palsy (RLNPBP), superior laryngeal nerve deficit (SLD), and other complications. Moreover, we analysed the total excision rate and indications to perform postoperative RAI therapy or other adjuvant treatments. Of the 111 patients recruited, 53 were on regular follow-up (FU) at our Centre. Complete data were available on disease-free survival, total survival, and recurrence. Of the remaining 58 patients, we could not obtain up-to-date information, and they were therefore considered lost to follow-up. After treatment, all patients received levothyroxine TSH suppressive therapy and were entrusted to the attending endocrinologists for follow-up treatment. All ADTC patients were considered high-risk and underwent periodic blood checks consisting of TSH, thyroglobulin, and antithyroglobulin antibodies, as well as a cervical ultrasonography (US) every 6–12 months, for an average period of 15 years (range 2–30 years).

## Results

### Population

A total of 111 patients were enrolled in our study, 74 women and 37 men. The median age is 45.8 ± 19.5 SD years of age. Women were in the age ranges of 19 to 93, and men ranging between 11 and 81 years, with women’s mean age of 46.4 ± 20.4 and men’s 44.4 ± 17.9. We enrolled and treated exclusively in the abovementioned centre 96 patients, while 15 had previous thyroid surgery in other Italian centres.

### Clinical History

Family history of cancer thyroid disease was present in 26/111 patients (23.42%), in particular 7 men (18.92%) and 19 women (25.68%). We identified 53 patients (47.75%) coming from areas known for endemic goitres; 17 are men (45.95%), and 36 are women (48.65%). Of our sample, 35 women (47.30%) were menopausal at the time of diagnosis. The clinical characteristics of the patients enrolled in the study are briefly summarized in **Table 1**.

**Table 1:** Clinical features of patients (n 111).

Clinical features	Value
**Sex *n* (%)** Female (F) Male (M)	74 (66.7%) 37 (33.3%)
**Age at surgery** Mean ± DS Median (range)	45.8 ± 19.5 46 (11–93)
**Pre-operative TSH** Mean ± DS Median (range)	1,97 ± 0,17 2,1 (0.0015–6.99)
**Preoperative fT_3_** Mean ± DS Median (range)	2.91 ± 0.07 3.1 (2.1–30)
**Preoperative fT_4_** Mean ± DS Median (range)	1.02 ± 0.18 1.2 (0.34–45.81)
**Presentation with** Lymph node metastases Locally advanced Distant metastases	76 (68.47%) 22 (19.82%) 7 (6.3%)
**Surgical treatment *n* (%)** TT + CCL TT + CUCL TT + CBCL UCL BCL	21 (18.9%) 70 (63.1%) 5 (4.5%) 10 (9.0%) 5 (4.5%)

### Clinical Presentation

At the time of diagnosis, 76/111 (68.5%) patients had lymph node metastases, and the presence of a cervical mass was the problem that initiated the diagnostic process, which then led to the discovery of DCTs.

In the other cases, at the time of diagnosis, 22/111 patients (19.8%) presented with a locally advanced tumour characterized by the involvement of adjacent anatomical structures. All of them presented with local symptomatology and thus were referred for clinical evaluation. In this group of 22 patients, 15 were women and 7 were men, respectively 18.92% and 21.62% of their groups. The most frequent symptoms were dysphonia (8/22 cases), dysphagia (3/22 cases), and cervical encumbrance sensation (19/22 cases).

For six out of 111 (5.4%) patients, the tumour was occasionally detected incidentally during clinical examination conducted for other reasons and they were asymptomatic at the time of diagnosis.

In our study, seven out of 111 (6.3%) patients were diagnosed with DCTs after clinical examinations for distant metastasis presence, initially unknown, but later identified as a repetition from DCT. The distant metastases included the following groups: bone metastasis of the mandible, liver metastasis, lung and liver metastasis, two isolated lung metastases, one multiple lung metastasis, and multiple bone metastasis of three ribs.

After preoperative blood tests, five out of 111 cases of reduced TSH levels were detected (normal range 0.15–3.5 U/ml); two out of 111 patients had increased TSH levels, while the remaining patients were within the normal range. Only four out of 111 patients were hypocalcemic, but three previously underwent TT at different Italian centres. Only a single patient was hypercalcemic.

### Treatment and Complications

Surgical procedures consisted of 21 (18.9%) TT and CCL, 70 (63.1%) TT and CUCL, 5 (4.5%) TT and CBCL, 10 (9.0%) UCL, and 5 (4.5%) BCL after previous thyroidectomy in other Italian centres. None of the patients enrolled in the study underwent hemithyroidectomy. In the case of local advanced tumours or distant metastases, patients received tailored treatments based on their individual clinical condition. For details, see **Table 2**.

**Table 2:** Detailed treatment received by patients in case of local advanced tumour or distance metastases. In brackets are the reported number of cases.

Clinical condition	Treatment
Complete unilateral vocal cord palsy (6)	Unilateral laryngeal nerve resection
Unilateral vocal cord hypomobility (3)	Laryngeal nerve preservation
Intraoperative evidence of nerve hypofunction with neuromonitoring in extensive tumour infiltration (1)	Unilateral laryngeal nerve resection
Laryngeal and tracheal massive infiltration (1)	The patient refused to undergo total laryngectomy and tracheal resection preoperatively. Surgical excision was therefore subtotal followed by radioiodine treatment.
Laryngeal minimal infiltration (1)	Thyroid nodules identified during routine carotid echo doppler: PTC infiltrating proximal tissues. During TT+CCL, evidence of millimetric laryngeal infiltration was left in place. Radioiodine treatment. In 5 months, the patient developed a massive neck mass, with extensive laryngeal invasion. The patient underwent total laryngectomy + BCL.
Lung metastasis (8)	One patient underwent atypical thoracoscopic lung resection, with metastasis excision. All the other patients received only adjuvant radioiodine treatment.
Mandibular metastasis (1)	Nontoxic thyroid goitre, not in regular follow-up. Clinical evaluation was requested for a mandibular lesion, which showed to be an FTC metastasis. The patient underwent TT+CBCL and mandibular resection. After radioiodine evidence of lung, clavicle, spine, femoral, and skull base metastasis.
Rib metastasis (1)	Three bone metastases in three different ribs at diagnosis. The patient underwent TT+CCL and rib resection.
Brain metastasis (1)	PTC, treated with TT+UCLC. After 11 months, the patient underwent contralateral CLC for tumour relapse. After 13 months, a brain metastasis was detected, followed by trans-cranial excision.
Liver metastasis (1)	Evidence of liver lesion during examinations for other reasons, FTC metastasis. The patient underwent TT+CCL, and after full recovery (2 months), she underwent robotic left hepatectomy.

The final histological examination on the samples obtained during surgery reported 9/111 (8.1%) cases of follicular carcinoma and 102/111 (91.9%) cases of papillary carcinoma.

After the surgery, 87/111 patients (78,3%) developed a temporary or persistent complication as summarized in **Table 3**.

**Table 3:** Post-surgical complications developed in our series.

TH	53/111	45 patients have total spontaneous recovery
PH	8/53	Patients have not recovered, needing persistent pharmacological support
RLNTUP	3/111	Full spontaneous recovery in about 6 months
RLNPUP	8/111	7 of them were “obliged” nerve resections, as shown in **Table 2**
RLNTBP	1/111	The patient underwent tracheostomy
SLD	1/111	Persistent nerve deficit
Hypoglossal nerve deficit	1/111	Total spontaneous recovery in 4 months
Unilateral spinal accessory deficit	2/111	Persistent nerve deficit
Brachial plexus deficit	1/111	Total spontaneous recovery in 3 months
Marginal mandibular branches of the facial nerve deficit	2/111	Total spontaneous recovery in about 6 months
Oedemas of the surgical wound	4/111	
Postoperative bleeding	1/111	Immediate revision surgery
Pulmonary embolism	1/111	Massive PE 3 weeks after TT. *Exitus*

TH, temporary hypocalcemia, PH, persistent hypocalcemia; RLNTUP, recurrent laryngeal nerve transient unilateral palsy; RLNTBP, recurrent laryngeal nerve transient bilateral palsy; RLNPUP, recurrent laryngeal nerve persistent unilateral palsy; RLNPBP, recurrent laryngeal nerve persistent bilateral palsy; SLD, superior laryngeal nerve deficit.

### Postsurgical Treatments and Follow-up

A total of 110 patients received adjuvant radioiodine therapy after surgery; only one patient died 3 weeks after surgery, as reported in **Table 3**. They underwent from one to five cycles of treatment, with a mean of 2.4 ± 1.3, and of 98 ± 19 mCu total radiation dose. Of the 111 patients in our cohort, 53 remained in regular FU at our centre.

The three out of 53 patients that underwent TT and CCL presented peculiar conditions, such as liver, lung, or rib metastasis. After thyroid surgery, they all received surgical treatment for metastatic lesions (left robotic hepatectomy, atypical lung resection, and rib removal).

Of a total of 53 patients, 46 underwent TT and CUCL, 5 of them with LN resection, and 4 underwent TT and CBCL, 2 of which with LN resection. Moreover, one of these four patients presented mandible metastasis as symptom onset. She underwent mandible resection, with total metastasis gross excision.

Only 8/53 (15.1%) (3 men and 5 women, mean age 69 years old) are deceased, while 45/53 (84.9%) are currently alive (16 men and 29 women, mean age 42 years old). Patients currently still alive have a mean FU of 165 ± 101.2 months.

A total of 39/45 (86.7%) never developed recurrences; they all underwent TT and CUCL. Serum Tg and Ab-Tg never showed an increase during the follow-up period. Neck ultrasound always resulted negative. Five out of 45 (11.1%) have developed relapse, and one (2.2%) had persistence. The persistence was subjected to the neck radiotherapy cycle (5550 Mbq). The five relapses included heterogeneous cases (**Table 4**).

**Table 4:** Clinical history and treatment of the five relapsed patients.

Patient 1	During radioiodine therapy, development of cervical lymph node metastasis (left V level). UCL + removal of a portion of infiltrated sternocleidomastoid.
Patient 2	Recurrence at the thyroid bed 1 year after surgery. Revision surgery, followed by external radiotherapy and radioiodine
Patient 3	V level lymph node metastasis from papillary carcinoma 12 years after surgery, treated with UCL.
Patient 4	Contralateral lymph node localization 11 months after TT+CUCL. He underwent a second UCL.
Patient 5	Contralateral lymph node localization 13 months after TT+CUCL. He underwent a second UCL and a new course of radioiodine therapy (130 mCu)

Three of eight patients deceased underwent TT and CCL. Three out of eight underwent TT and CUCL, and two out of eight underwent TT and CBCL. They had a mean survival of 47 months, ranging from 14 days to 151 months. All these patients presented distinctive characteristics as shown in **Table 5**.

**Table 5:** Clinical history and treatment of the eight deceased patients.

Patient 1	Thyroid cartilage infiltration, revealed during TT. Adjuvant radioiodine therapy alone to preserve the organ. After 8 months, massive cervical metastasis associated with local laryngeal progression, which led to total laryngectomy. The patient died 15 months later secondary to other medical conditions.
Patient 2	Left vocal cord palsy at diagnosis. TT+CCL+ resection of the left lower laryngeal recurrent nerve. The patient died 22 months following another clinical condition.
Patient 3	Macroscopic laryngo-tracheal tumour penetration at the time of diagnosis, but the patient refused total laryngectomy and tracheal resection. Surgical excision was subtotal (TT+CCL) followed by radioiodine treatment. The patient died 5 months after secondary to another clinical condition.
Patient 4	TT+CCL. The patient died at 14 days for pulmonary embolism.
Patient 5	TT+CUCL. Local disease recurrence, in the thyroid bed and in an area of oesophageal infiltration and a delayed onset lymph node metastasis. The patient died 151 months after due to tumour progression.
Patient 6	TT+CUCL. Multiple lung and bone metastases at the time of diagnosis. The patient died 111 months after due to tumour progression.
Patient 7	TT+CBCL. Five months later, the patient showed a local macroscopic recurrence with the involvement of trachea and larynx, treated with a total laryngectomy. The patient also underwent therapies with tyrosine kinase inhibitors but died 12 months later.
Patient 8	TT+CBCL. Multiple lung metastases at the time of diagnosis. Progressive development of bone and brain metastasis. The patient died 55 months after due to tumour progression.

In our series, only one patient refused surgery and was therefore not included in the above discussion. He underwent treatment with lenvatinib and is now in good clinical condition with tumour regression 20 months after diagnosis. The 45 patients still alive and in regular follow-up are in good clinical condition. During a telephone interview specifically for the present study, they reported living a normal life with good quality of life, taking into account age and other pathologies. They are all in thyroxine suppression therapy and present a mean TSH level of 0.54 µIU/ml (range 0.0015–3.61 µU/ml), mean Tg 0.07 ng/ml (range 0.04– 0.59 ng/ml), and Ab anti Tg 11.26 IU/ml (0–20 IU/ml), and all have neck ultrasound negative for thyroid bed and lymph node recurrence.

## Discussion

There is no standard definition for ADTC in the literature. In the ATA guidelines published in 2016 (9), there is a session entitled “DTC: Long-Term Management and Advanced Cancer Management Guidelines,” but the definition of ADTC is missing. In our series, we defined ADTC according to Russel et al. (5): invasion of thyroid adjacent structures (local invasion), laterocervical lymph nodes, metastasis, distant metastases, and more than one of the previously mentioned characteristics simultaneously.

The first consideration in the analysis of the present study is treatment. The different therapeutic choices have been tailored to the characteristics of each patient, and this approach has led to encouraging results: 84.9% of the 45 patients with available follow-up are currently alive, with a median FU > 10 years, i.e., 165 months (range 12–345 months). All of them were in the group with regional lymph node metastases, more in detail, eight with locally advanced tumours. Among these 45, four (11.1%) have developed relapse, in all cases localized in the neck, that was subsequently successfully treated by re-surgery and adjuvant therapy. None of the 45 patients developed distant metastases. Two of these relapses had at the time of their first intervention one RLN infiltration and the other tracheal cartilage infiltration. Only one (2.2%) has persistence in the thyroid bed, without disease progression. For relapse and persistence, the histological result was PTC. Of the eight patients who died, only four died of neoplastic causes, two of them after more than 10 years, 151 months, and 111 months, both with histological results of PTC. The two patients with the worst evolution were, one, already diagnosed with multiple lung and bone metastases for follicular carcinoma, and the other, showed trachea and larynx involvement for papillary carcinoma.

According to literature (10, 11), local invasiveness, albeit with a limited number of local ADTC in our case series, had a greater impact on survival than cervical lymph node involvement. However, DTCs are generally slow-evolving tumours, even if advanced. This aspect should be the first to be considered in the therapeutic choice of ADTC. With regard to complications associated with surgery, we observed a higher percentage of definitive hypoparathyroidism and persistent RLNUP in thyroidectomies performed for diseases other than ADTC carcinoma (12, 13). This may be related to the fact that central neck dissection, always performed in all patients included in this study, is a well-known risk factor for persistent hypoparathyroidism. Regarding the definitive RLNUP, the locally advanced situation of the tumour, with the involvement of the RLN in 10 cases, preoperative palsy of the vocal cord and intraoperative nerve infiltration in 6, and intraoperative infiltration in 4 cases, leading to sacrifice in one, has increased the number of definitive vocal cord paralysis. In the case of locally advanced tumours (LADTC), the most commonly involved structure in our experience was the recurrent laryngeal inferior nerve (RLN), more specifically in 10 cases.

In agreement with the literature, treatment of the infiltrated RLN was based on the knowledge of preoperative vocal function (14, 15), magnitude of neural invasion, and occurrence of loco-regional or distant metastases not surgically removable. There is a general agreement that attempts should be made to preserve RLN when preoperative vocal cord function is normal, and intraoperatively it appears only to be superficially invaded. The location of the RLN invasion should be taken into account in the decision to preserve it or not: resection should be performed when neural invasion occurs near the point where the RLN enters the larynx. This is because an incomplete resection of the tumour at this point can lead to the progression of the tumour along the nerve and its spread into the larynx, with subsequent indications for laryngectomy (5). In addition to the location, the extent of neural invasion should influence intraoperative decision-making. When only the epineurium is infiltrated, a shave excision may be performed (14–16). Even if the nerve shaving may cause partial layer resection, most nerves demonstrate a long-term recovery of neural function (14). On the contrary, if the perineurium, endoneurium, and neural fibres are invaded, the resection should be considered. In this case, the simple shaving of the nerve without the complete removal of the tumour may be considered, as we did in the three cases abovementioned, that is, if adjuvant RAI therapy may treat the residual neoplastic tissue (14, 16–18). When intraoperative neuromonitoring (IONM) is routinely used during surgery, it is important to evaluate the possibility of proximally stimulating the nerve (18): the ability to stimulate the proximal side of an infiltrated RLN suggests some maintenance of neural function, even if paralysis of the vocal cord is documented preoperatively. As reported by Kamani et al., 60% of invaded nerves can be stimulated with IONM (19) and the resection of invaded RLN with preoperative paralysis of the vocal cord, according to some authors (20), may worsen glottic function. For this reason, the ability to stimulate the proximal segment of the invaded nerve may be a parameter for intraoperative decision to preserve or not the nerve (14). A very important aspect to consider about the treatment of an infiltrated RLN is that resecting or preserving it would not seem to change the prognosis (14, 17, 18, 21–23). For this reason, if possible, it would be best to preserve the sRLN, because the integrity of the nerve corresponds to the good functioning of the vocal cord, which has a great impact on the quality of life. A negative impact on the quality of life may occur when there is an infiltration of viscera adjacent to the thyroid, such as airways (larynx and/or trachea), oesophagus, and vascular structures. In our experience, among ADCT not lost at follow-up, five had invasions of the airway, two had laryngeal invasion, and three had tracheal invasion. One patient with laryngeal infiltration refused laryngectomy and died after 25. Another patient had infiltration of the larynx and the first two tracheal rings. She underwent laryngectomy 5 months after TT, and she died 12 months later. The others are alive respectively after 37, 77, and 101 months, without resecting trachea. They were treated with tracheal shaving during TT and CCL as well as postoperative RAI, referring to good quality of life. In the presence of visceral structure invasion, the objective of surgery should be to remove the majority of the tumour mass. ATA guidelines recommend surgery for tumours invading the upper aerodigestive tract along with ^131^I (RAI) and/or external beam radiation therapy (ERBT) (9, 24, 25). In some patients, surgery may represent a viable cure effort, while in others it represents a palliative approach to reduce symptoms such as asphyxia and haemoptysis (26, 27). In the case of tracheal infiltration, surgical techniques depend on the type of tumoral invasion. The extent of tracheal invasion has been classified by Shin, as well as others (21, 27, 28), in four stages: perichondrial invasion with adherence to the trachea (stage I), cartilaginous invasion but without mucosal involvement (stage II), mucosal infiltration (stage III), and extension into the lumen (stage IV). In the suspicion of tracheal involvement, besides CT scan, preoperative bronchoscopy is mandatory, to assess mucosal intraluminal involvement. Shave resection of the airway may be appropriated in cases of limited cartilage invasion, involving tiny segments. In the presence of more extensive or intraluminal tumour infiltration, tracheal resection is suggested (21, 29). In the case of larynx infiltration, shave excision is possible when the tumour does not extend into the larynx. Otherwise, more aggressive treatment, such as laryngectomy, may be required (27). In our series, patients with laterocervical lymph node metastases, not lost at follow-up, were 44. All had a functional cervical dissection, including II, III, IV, and V levels. Five of them had recurrences, and 32 were alive following a mean follow-up of 210 months. Treatment of distant metastases should consider that both morbidity and mortality are increased in patients with distant metastases and that individual prognosis is influenced by a number of factors, including histology of the primary tumour, distribution and number of metastatic sites (e.g., brain, bone, lung), age at diagnosis, and RAI avidity (30–38). Treatment of a specific metastasis should be grounded on patient status and on the presence of other sites of disease (39–42). According to ATA (9), the sequence of therapeutic choices for the treatment of metastatic disease should be in order:

*i)* surgical excision of loco-regional metastasis in potentially curable patients;
*ii)* ^131^I therapy for RAI-responsive disease;
*iii)*ERBT or other directed treatment modalities for metastases;
*iv)* TSH-suppressive thyroid hormone therapy for patients, with stable or slowly progressive asymptomatic disease;
*v)* systematic therapy with kinase inhibitors, especially for patients with significantly progressive macroscopic refractory disease.

In the management of the patient with lung metastases, therapeutic decisions should consider the following: size of metastatic lesions (i.e., macro-nodular detected by chest radiography, micro-nodular detected by CT, lesions below CT resolution); avidity for RAI; response to previous RAI therapy; stability or not of metastatic lesions. In view of the high rate of the observed complete remission, lung micro-metastases should be treated with RAI therapy and eventually be repeated every 6–12 months, if the disease continues to concentrate RAI and respond clinically (41, 42). Also, radioiodine-avid macro-nodular metastases should be treated with RAI when objective benefit is demonstrated (i.e., decrease in the size of the lesions, decreasing Tg). In the latter, however, complete remission is uncommon and survival remains poor. It is also worth considering the risks associated with repeated doses of RAI such as bone marrow suppression or pulmonary pneumonitis and fibrosis. Solitary lung metastases could be resected *via* surgery, even though the potential benefits compared with the risk of surgery remain unclear. With regard to bone metastasis, RAI therapy may be considered for those that are iodine-avid. In these cases, the therapy has been associated with improved survival, although RAI is rarely curative. Regardless, some patients with RAI-avid bone metastases may still benefit from this therapy (35–37). These patients may also be considered for directed therapy in cases where metastases are visible on anatomical imaging. Directed therapies include surgery, EBRT, and other focal treatment modalities discussed below. Systemic therapy with bone-targeted agents may also be considered in these patients. The therapeutic approaches to brain and liver metastases are similar to those previously mentioned for lungs and bone metastases (9). As mentioned, several focal treatments other than surgery, for lung, bone, liver, and brain DTC metastases, are available. In certain patients, these treatment modalities, alternative to surgery, may be effective for local tumour control as first-line treatment with similar efficacy to that of surgical resection. In general, such treatments may be indicated in case of high anaesthesiologic risk, lung metastasis in patients with insufficient respiratory reserve, multiple previous surgical resections, local recurrence after surgery, and refusal of additional surgery. In our experience, we treated seven metastatic patients: two had pulmonary metastases, three had bone metastases, one had liver metastases, and one had brain metastases. Of the two patients with lung metastases, one was a young patient for whom the diagnosis of DTC was made through the removal of a solitary pulmonary nodule, with pathological examination showing PTC metastasis as a result. Then, only a single sub-centimetre nodule was found during neck ultrasound and she underwent total thyroidectomy with central neck dissection. After these two interventions, she underwent RAI with no evidence of further lesions. She remains disease free at her 89-month follow-up. The other one showed multiple pulmonary areas of uptake at post-thyroidectomy ^131^I total body scan, after total thyroidectomy for follicular carcinoma. During a chest CT, she showed multiple micro-metastases. She underwent three cycles of RAI therapy. She is alive and persistent but reduced for many metastases at the 125-month follow-up. Of the three patients with bone metastases, two were treated: patients with ADTC are treated by RAI. The dose of RAI, the number of treatments, and the time between treatments should be personalized depending on unique patient characteristics such as disease response to therapy, patient’s age, and the onset of side effects (i.e., radiation lung damage, bone-marrow suppression, and salivary gland damage). Therapy should be repeated at least 6–12 months after the previous treatment (5). Finally, systemic therapy may be considered in patients with locally advanced and/or metastatic RAI refractory disease not suitable for local therapies with the purpose to reduce tumour growth and/or metastatic spread (43). The first-line systemic therapy is represented by the multikinase inhibitors lenvatinib and sorafenib, approved by FDA and EMA (44–49). In our series, we have two patients enrolled by endocrinologists for systemic therapy with lenvatinib, as described before: one for local advanced and metastatic disease who refused intervention and the other for multiple recurrent metastatic DTCs (4). Nowadays, traditional chemotherapy is indicated only in selected cases: when multikinase inhibitors are ineffective or cannot be used. If ^131^I-refractory, metastatic patients should be maintained only with TSH-suppressive thyroid hormone therapy if they are asymptomatic or stable or have a minimally progressive disease without clinically significant complications. These patients need serial radiographic imaging every 3–12 months (9).

## Conclusions

In the case of locally and/or metastatic ADTC, there are many therapeutic options available. The best treatment modality must take into consideration various aspects related to the patient and the pathology at hand. Moreover, the benefits of surgical excision of a slow-evolving tumour, such as DTC, in terms of local control, risk of persistence/recurrence, and survival, must always be carefully weighed against the morbidity of surgery, which may require aggressive resection of extrathyroidal tissues. For these reasons, a single standardized approach for local and/or metastatic ADTC could not be established, but rather a tailored treatment must be defined for each patient. The best treatment certainly requires a multidisciplinary group, consisting of an endocrinologist, an expert endocrine surgeon, an otolaryngologist, a radiologist, a nuclear medicine doctor, and an oncologist, all operating in a high-volume centre. Multicentre studies from high-volume centres would be desirable, to optimize the choice of various therapeutic options discussed before, and to offer the best tailored treatment to the patients affected with locally and/or metastatic ADTC.

## Data Availability Statement

The original contributions presented in the study are included in the article/supplementary material. Further inquiries can be directed to the corresponding author.

## Ethics Statement

Ethical review and approval was not required for the study on human participants in accordance with the local legislation and institutional requirements. The patients/participants provided their written informed consent to participate in this study.

## Author Contribution

Conceptualization: LP; methodology: LP, AB and MB; validation PL, LC and AS; investigation: AB, EB and LP; writing—original draft preparation: AB, LP, MB and EL; writing—review and editing: MB, EL, EF, CP, CR and GF. All authors have read and agreed to the published version of the manuscript. All authors listed have made a substantial, direct, and intellectual contribution to the work and approved it for publication.

## Conflict of Interest

The authors declare that the research was conducted in the absence of any commercial or financial relationships that could be construed as a potential conflict of interest.

## Publisher’s Note

All claims expressed in this article are solely those of the authors and do not necessarily represent those of their affiliated organizations, or those of the publisher, the editors and the reviewers. Any product that may be evaluated in this article, or claim that may be made by its manufacturer, is not guaranteed or endorsed by the publisher.

## References

[1] Van Den Heede K Tolley NS Di Marco AN Palazzo FF. Differentiated Thyroid Cancer: A Health Economic Review. Cancers (Basel). (2021) 13(9):2253. doi: 10.3390/cancers13092253 34067214 PMC8125846

[2] Araque KA Gubbi S Klubo-Gwiezdzinska J. Updates on the Management of Thyroid Cancer. Horm Metab Res (2020) 52(8):562–77. doi: 10.1055/a-1089-7870 Orphanet PMC7415555 32040962

[3] Differentiated Thyroid Carcinoma . Available at: https://www.orpha.net/consor/cgi-bin/OC_Exp.php?Lng=GB&Expert=146 (Accessed 2 April 2022).

[4] Russell MD Kamani D Randolph GW. Modern Surgery for Advanced Thyroid Cancer: A Tailored Approach. Gland Surg (2020) 9(Suppl 2):S105–19. doi: 10.21037/gs.2019.12.16 PMC7044078 32175251

[5] Pacini F Basolo F Bellantone R Boni G Cannizzaro MA De Palma M. Italian Consensus on Diagnosis and Treatment of Differentiated Thyroid Cancer: Joint Statements of Six Italian Societies. J Endocrinol Invest. (2018) 41(7):849–76. doi: 10.1007/s40618-018-0884-2 29729004

[6] Ulisse S Baldini E Lauro A Pironi D Tripodi D Lori E. Papillary Thyroid Cancer Prognosis: An Evolving Field. Cancers (Basel). (2021) 13(21):5567. doi: 10.3390/cancers13215567 34771729 PMC8582937

[7] Matsuzu K Sugino K Masudo K Nagahama M Kitagawa W Shibuya H. Thyroid Lobectomy for Papillary Thyroid Cancer: Long-Term Follow-Up Study of 1,088 Cases. World J Surg (2014) 38(1):68–79. doi: 10.1007/s00268-013-2224-1 24081532

[8] Adam MA Pura J Gu L Dinan MA Tyler DS Reed SD. Extent of Surgery for Papillary Thyroid Cancer is Not Associated With Survival: An Analysis of 61,775 Patients. Ann Surg (2014) 260(4):601–7. doi: 10.1097/SLA.0000000000000925 PMC4532384 25203876

[9] Haugen BR Alexander EK Bible KC Doherty GM Mandel SJ Nikiforov YE. 2015 American Thyroid Association Management Guidelines for Adult Patients With Thyroid Nodules and Differentiated Thyroid Cancer: The American Thyroid Association Guidelines Task Force on Thyroid Nodules and Differentiated Thyroid Cancer. Thyroid. (2016) 26(1):1–133. doi: 10.1089/thy.2015.0020 26462967 PMC4739132

[10] Lirov R Worden FP Cohen MS. The Treatment of Advanced Thyroid Cancer in the Age of Novel Targeted Therapies. Drugs. (2017) 77(7):733–45. doi: 10.1007/s40265-017-0733-1 PMC5683961 28361210

[11] Jung YS Oh CM Kim Y Jung KW Ryu J Won YJ. Long-Term Survival of Patients With Thyroid Cancer According to the Methods of Tumor Detection: A Nationwide Cohort Study in Korea. PloS One (2018) 13(4):e0194743. doi: 10.1371/journal.pone.0194743 29659584 PMC5901988

[12] De Pasquale L Sartori PV Vicentini L Beretta E Boniardi M Leopaldi E. Necessity of Therapy for Post-Thyroidectomy Hypocalcaemia: A Multi-Centre Experience. Langenbecks Arch Surg (2015) 400(3):319–24. doi: 10.1007/s00423-015-1292-0 25749741

[13] Zhang D Pino A Caruso E Dionigi G Sun H. Neural Monitoring in Thyroid Surgery is Here to Stay. Gland Surg (2020) 9(Suppl 1):S43–6. doi: 10.21037/gs.2019.10.24 PMC6995897 32055497

[14] Wu CW Dionigi G Barczynski M Chiang FY Dralle H Schneider R. International Neuromonitoring Study Group Guidelines 2018: Part II: Optimal Recurrent Laryngeal Nerve Management for Invasive Thyroid Cancer-Incorporation of Surgical, Laryngeal, and Neural Electrophysiologic Data. Laryngoscope. (2018) 128(Suppl 3):S18–27. doi: 10.1002/lary.27360 30291765

[15] Russell MD Kamani D Randolph GW. Surgical Management of the Compromised Recurrent Laryngeal Nerve in Thyroid Cancer. Best Pract Res Clin Endocrinol Metab (2019) 33(4):101282. doi: 10.1016/j.beem.2019.05.006 31230919

[16] Schneider R Randolph G Dionigi G Barczyński M Chiang FY Triponez F. Prospective Study of Vocal Fold Function After Loss of the Neuromonitoring Signal in Thyroid Surgery: The International Neural Monitoring Study Group's POLT Study. Laryngoscope. (2016) 126(5):1260–6. doi: 10.1002/lary.25807 26667156

[17] Kihara M Miyauchi A Yabuta T Higashiyama T Fukushima M Ito Y. Outcome of Vocal Cord Function After Partial Layer Resection of the Recurrent Laryngeal Nerve in Patients With Invasive Papillary Thyroid Cancer. Surgery. (2014) 155(1):184–9. doi: 10.1016/j.surg.2013.06.052 24646959

[18] Lang BH Lo CY Wong KP Wan KY. Should an Involved But Functioning Recurrent Laryngeal Nerve be Shaved or Resected in a Locally Advanced Papillary Thyroid Carcinoma? Ann Surg Oncol (2013) 20(9):2951–7. doi: 10.1245/s10434-013-2984-8 23636513

[19] Kamani D Darr EA Randolph GW. Electrophysiologic Monitoring Characteristics of the Recurrent Laryngeal Nerve Preoperatively Paralyzed or Invaded With Malignancy. Otolaryngol Head Neck Surg (2013) 149(5):682–8. doi: 10.1177/0194599813504735 24046274

[20] Chi SY Lammers B Boehner H Pohl P Goretzki PE. Is it Meaningful to Preserve a Palsied Recurrent Laryngeal Nerve? Thyroid. (2008) 18(3):363–6. doi: 10.1089/thy.2007.0124 18303959

[21] Shindo ML Caruana SM Kandil E McCaffrey JC Orloff LA Porterfield JR. Management of Invasive Well-Differentiated Thyroid Cancer: An American Head and Neck Society Consensus Statement. AHNS Consensus Statement. Head Neck. (2014) 36(10):1379–90. doi: 10.1002/hed.23619 24470171

[22] Nishida T Nakao K Hamaji M Kamiike W Kurozumi K Matsuda H. Preservation of Recurrent Laryngeal Nerve Invaded by Differentiated Thyroid Cancer. Ann Surg (1997) 226(1):85–91. doi: 10.1097/00000658-199707000-00012 9242342 PMC1190911

[23] Falk SA McCaffrey TV. Management of the Recurrent Laryngeal Nerve in Suspected and Proven Thyroid Cancer. Otolaryngol Head Neck Surg (1995) 113(1):42–8. doi: 10.1016/s0194-5998(95)70143-5 7603720

[24] Kim JW Roh JL Gong G Cho KJ Choi SH Nam SY. Treatment Outcomes and Risk Factors for Recurrence After Definitive Surgery of Locally Invasive Well-Differentiated Papillary Thyroid Carcinoma. Thyroid. (2016) 26(2):262–70. doi: 10.1089/thy.2015.0433 26566765

[25] McCaffrey JC. Evaluation and Treatment of Aerodigestive Tract Invasion by Well-Differentiated Thyroid Carcinoma. Cancer Control. (2000) 7(3):246–52. doi: 10.1177/107327480000700304 10832111

[26] Musholt TJ Musholt PB Behrend M Raab R Scheumann GF Klempnauer J. Invasive Differentiated Thyroid Carcinoma: Tracheal Resection and Reconstruction Procedures in the Hands of the Endocrine Surgeon. Surgery. (1999) 126(6):1078–88. doi: 10.1067/msy.2099.102267 10598191

[27] Czaja JM McCaffrey TV. The Surgical Management of Laryngotracheal Invasion by Well-Differentiated Papillary Thyroid Carcinoma. Arch Otolaryngol Head Neck Surg (1997) 123(5):484–90. doi: 10.1001/archotol.1997.01900050030003 9158394

[28] Shin DH Mark EJ Suen HC Grillo HC. Pathologic Staging of Papillary Carcinoma of the Thyroid With Airway Invasion Based on the Anatomic Manner of Extension to the Trachea: A Clinicopathologic Study Based on 22 Patients Who Underwent Thyroidectomy and Airway Resection. Hum Pathol (1993) 24(8):866–70. doi: 10.1016/0046-8177(93)90136-5 8375857

[29] Merdad M Eskander A Kroeker T Freeman JL. Predictors of Level II and Vb Neck Disease in Metastatic Papillary Thyroid Cancer. Arch Otolaryngol Head Neck Surg (2012) 138(11):1030–3. doi: 10.1001/2013.jamaoto.393 23165376

[30] Bernier MO Leenhardt L Hoang C Aurengo A Mary JY Menegaux F. Survival and Therapeutic Modalities in Patients With Bone Metastases of Differentiated Thyroid Carcinomas. J Clin Endocrinol Metab (2001) 86(4):1568–73. doi: 10.1210/jcem.86.4.7390 11297585

[31] Chiu AC Delpassand ES Sherman SI. Prognosis and Treatment of Brain Metastases in Thyroid Carcinoma. J Clin Endocrinol Metab (1997) 82(11):3637–42. doi: 10.1210/jcem.82.11.4386 9360519

[32] Ronga G Filesi M Montesano T Di Nicola AD Pace C Travascio L. Lung Metastases From Differentiated Thyroid Carcinoma. A 40 Years' Experience. Q J Nucl Med Mol Imaging. (2004) 48(1):12–9. 15194999

[33] Lin JD Chao TC Chou SC Hsueh C. Papillary Thyroid Carcinomas With Lung Metastases. Thyroid. (2004) 14(12):1091–6. doi: 10.1089/thy.2004.14.1091 15650364

[34] Shoup M Stojadinovic A Nissan A Ghossein RA Freedman S Brennan MF. Prognostic Indicators of Outcomes in Patients With Distant Metastases From Differentiated Thyroid Carcinoma. J Am Coll Surg (2003) 197(2):191–7. doi: 10.1016/S1072-7515(03)00332-6 12892796

[35] Zettinig G Fueger BJ Passler C Kaserer K Pirich C Dudczak R. Long-Term Follow-Up of Patients With Bone Metastases From Differentiated Thyroid Carcinoma – Surgery or Conventional Therapy? Clin Endocrinol (Oxf). (2002) 56(3):377–82. doi: 10.1046/j.1365-2265.2002.01482.x 11940050

[36] Pittas AG Adler M Fazzari M Tickoo S Rosai J Larson SM. Bone Metastases From Thyroid Carcinoma: Clinical Characteristics and Prognostic Variables in One Hundred Forty-Six Patients. Thyroid. (2000) 10(3):261–8. doi: 10.1089/thy.2000.10.261 10779141

[37] Schlumberger M Challeton C De Vathaire F Travagli JP Gardet P Lumbroso JD. Radioactive Iodine Treatment and External Radiotherapy for Lung and Bone Metastases From Thyroid Carcinoma. J Nucl Med (1996) 37(4):598–605. 8691248

[38] Dinneen SF Valimaki MJ Bergstralh EJ Goellner JR Gorman CA Hay ID. Distant Metastases in Papillary Thyroid Carcinoma: 100 Cases Observed at One Institution During 5 Decades. J Clin Endocrinol Metab (1995) 80(7):2041–5. doi: 10.1210/jcem.80.7.7608252 7608252

[39] Pak H Gourgiotis L Chang WI Guthrie LC Skarulis MC Reynolds JC. Role of Metastasectomy in the Management of Thyroid Carcinoma: The NIH Experience. J Surg Oncol (2003) 82(1):10–8. doi: 10.1002/jso.10189 12501164

[40] Kitamura Y Shimizu K Nagahama M Sugino K Ozaki O Mimura T. Immediate Causes of Death in Thyroid Carcinoma: Clinicopathological Analysis of 161 Fatal Cases. J Clin Endocrinol Metab (1999) 84(11):4043–9. doi: 10.1210/jcem.84.11.6115 10566647

[41] Ilgan S Karacalioglu AO Pabuscu Y Atac GK Arslan N Ozturk E. Iodine-131 Treatment and High-Resolution CT: Results in Patients With Lung Metastases From Differentiated Thyroid Carcinoma. Eur J Nucl Med Mol Imaging. (2004) 31(6):825–30. doi: 10.1007/s00259-004-1460-x 14762699

[42] Hod N Hagag P Baumer M Sandbank J Horne T. Differentiated Thyroid Carcinoma in Children and Young Adults: Evaluation of Response to Treatment. Clin Nucl Med (2005) 30(6):387–90. doi: 10.1097/01.rlu.0000162602.48653.54 15891289

[43] Baldini E Presutti D Favoriti P Santini S Papoff G Tuccilli C. *In Vitro* and *In Vivo* Effects of the Urokinase Plasminogen Activator Inhibitor WX-340 on Anaplastic Thyroid Cancer Cell Lines. Int J Mol Sci (2022) 23(7):3724. doi: 10.3390/ijms23073724 35409084 PMC8999125

[44] Elia G Ferrari SM Ragusa F Paparo SR Mazzi V Ulisse S. Advances in Pharmacotherapy for Advanced Thyroid Cancer of Follicular Origin (PTC, FTC). New Approved Drugs and Future Therapies. Expert Opin Pharmacother. (2022) 23(5):599–610. doi: 10.1080/14656566.2022.2030704 35038965

[45] Gabillard JC Ulisse S Baldini E Sorrenti S Cremet JY Coccaro C. Aurora-C Interacts With and Phosphorylates the Transforming Acidic Coiled-Coil 1 Protein. Biochem Biophys Res Commun (2011) 408(4):647–53. doi: 10.1016/j.bbrc.2011.04.078 21531210

[46] Ferrari SM Politti U Spisni R Materazzi G Baldini E Ulisse S. Sorafenib in the Treatment of Thyroid Cancer. Expert Rev Anticancer Ther (2015) 15(8):863–74. doi: 10.1586/14737140.2015.1064770 26152651

[47] Ferrari SM Bocci G Di Desidero T Elia G Ruffilli I Ragusa F. Lenvatinib Exhibits Antineoplastic Activity in Anaplastic Thyroid Cancer *In Vitro* and *In Vivo* . Oncol Rep (2018) 39(5):2225–34. doi: 10.3892/or.2018.6306 29517103

[48] Baldini E Tuccilli C Prinzi N Sorrenti S Antonelli A Gnessi L. Effects of Selective Inhibitors of Aurora Kinases on Anaplastic Thyroid Carcinoma Cell Lines. Endocr Relat Cancer. (2014) 21(5):797–811. doi: 10.1530/ERC-14-0299 25074669

[49] Baldini E Tuccilli C Pironi D Catania A Tartaglia F Di Matteo FM. Expression and Clinical Utility of Transcription Factors Involved in Epithelial-Mesenchymal Transition During Thyroid Cancer Progression. J Clin Med (2021) 10(18):4076. doi: 10.3390/jcm10184076 34575184 PMC8469282

